# Safety, Tolerability, and Effect of Nusinersen Treatment in Ambulatory Adults With 5q-SMA

**DOI:** 10.3389/fneur.2021.650535

**Published:** 2021-05-20

**Authors:** Bakri Elsheikh, Steven Severyn, Songzhu Zhao, David Kline, Matthew Linsenmayer, Kristina Kelly, Marco Tellez, Amy Bartlett, Sarah Heintzman, Jerry Reynolds, Gary Sterling, Tristan Weaver, Kiran Rajneesh, Stephen J. Kolb, W. David Arnold

**Affiliations:** ^1^Department of Neurology, The Ohio State University Wexner Medical Center, Columbus, OH, United States; ^2^Department of Anesthesiology, The Ohio State University Wexner Medical Center, Columbus, OH, United States; ^3^Department of Biomedical Informatics and Center for Biostatistics, The Ohio State University, Columbus, OH, United States; ^4^Assistive Technology Department, The Ohio State University Wexner Medical Center, Columbus, OH, United States

**Keywords:** ambulatory, spinal muscular atrophy, nusinersen, motor unit, reinnervation

## Abstract

**Objective:** To determine the safety and tolerability of nusinersen treatment in ambulatory adults with spinal muscular atrophy (SMA) and investigate the treatment effect on muscle strength, physical function, and motor unit physiology.

**Methods:** Individuals aged 18 years or older with genetically confirmed 5q SMA, three or more copies of the SMN2 gene, and the ability to ambulate 30 feet were enrolled. Safety outcomes included the number of adverse events and serious adverse events, clinically significant vital sign or laboratory parameter abnormalities. Outcome assessments occurred at baseline (prior to the first dose of nusinersen) and then 2, 6, 10, and 14 months post-treatment.

**Results:** Six women, seven men (mean age: 37 ± 11, range: 18–59 years) were included for analyses. The most common side effects were headache and back pain, but overall procedures and treatments were well-tolerated. No serious adverse events were reported. Maximal Voluntary Isometric Muscle Contraction Testing (MVICT) and 6-min walk test (6MWT) both showed overall stability with significant increases at 2, 6, and 10 months for the 6MWT. More consistent significant treatment effects were noted on the Hammersmith Functional Motor Scale Expanded, SMA-Functional Rating Scale, and forced vital capacity. Treatment resulted in progressively increased ulnar compound muscle action potential and average single motor unit potential amplitudes, but motor unit number estimation remained stable.

**Conclusions:** Nusinersen treatment is safe and well-tolerated in ambulatory adults with SMA. Treatment resulted in improved motor function and electrophysiological findings suggest that this improvement may be occurring *via* improved motor unit reinnervation capacity.

## Introduction

Spinal muscular atrophy (SMA) is an autosomal recessive motor neuron disorder caused by homozygous loss of function of the SMN1 gene (1). In SMA patients, full-length SMN protein is produced by a second gene, SMN2, but only in small amounts which are insufficient for normal neuromuscular function (2–6). Nusinersen (Spinraza) is an intrathecally-delivered therapy that increases full-length SMN protein production from the SMN2 gene. Nusinersen was approved for all types of SMA, but approval was primarily based on evidence in infants and children ages 2–15 (7–9). There is emerging data supporting the use of nusinersen in adults with SMA (10–17). However, there is a limited understanding of how delayed or late SMN restoration may result in improved function. Although ambulatory adults have a milder phenotype, the majority have significant weakness compared to age and gender matched controls (18). Limited data from prior studies suggest progressive strength and functional decline over time in all SMA participants including adults (19). However, data on long-term longitudinal changes in several of the standard outcome measures used in ambulatory adults is scant (19, 20).

We conducted a single-center, open-label study to evaluate the safety, tolerability and effect of nusinersen treatment in ambulatory SMA adults. As there are no prior studies that have investigated motor unit responses to nusinersen treatment in ambulatory adults with SMA, one of the major goals of our study was to interrogate the electrophysiological effects on motor unit function. Additionally, we assessed the longitudinal change in common outcome measures over 10 years for participants enrolled in a prior study SMA trial: a prospective, double-blind, placebo-controlled trial of valproic acid in ambulatory adults with spinal muscular atrophy (VALIANT) (20).

## Materials and Methods

### Study Design

This was a prospective, open- label, observational study conducted at the Ohio State University Wexner Medical Center. The study was approved by the institutional review board. Written informed consent was obtained before enrollment. Study visits were conducted between 06/2017 and 01/2020.

### Study Population

Inclusion criteria included: age ≥ 18, genetic confirmation of 5q SMA, ≥3 SMN2 copies, ability to walk 30 feet, and insurance approval for nusinersen or qualification for free drug program. Exclusion criteria included: spinal disease that would preclude intrathecal nusinersen delivery, history of bacterial meningitis or encephalitis, and use of investigational drug treatment for SMA in the last 6 months.

### Study Overview

Participants completed a screening visit within 4 weeks of starting nusinersen treatment to determine eligibility for participation. Eligible participants completed loading intrathecal nusinersen treatment on day 1, 15, 29, and 60 followed by maintenance doses every 4 months. Each dose was 12 mg, delivered *via* fluoroscopy-guided lumbar puncture using a 25 gauge Quincke spinal needle. Repeated assessments were completed within 2 weeks after nusinersen administration at 2, 6, 10, and 14 months. We also enrolled eight subjects who previously participated in the VALIANT trial to determine the long-term changes in outcome measures over the 10 years between VALIANT and the pre-nusinersen treatment assessments (20).

### Outcome Measures

The primary outcome was muscle strength change from baseline to 14 months as quantified by a Maximal Voluntary Isometric Muscle Contraction (MVICT) average score derived from 5 muscle groups tested bilaterally (10 total: bilateral elbow flexion and extension, hand grip and knee flexion and extension) (18, 20, 21). This outcome was chosen due to the fact that it was most reliable in our prior study (18). MVICT was performed using the Quantitative Muscle Assessment (QMA) system (Aeverl Medical LLC, Gainesville, Georgia). Patient and strap positioning for elbow flexion/extension and knee flexion/extension was performed as previously reported (21). Grip strength was measured with the elbow flexed to 90 degrees while the shoulder, forearm and wrist were in a neutral position; if the bicep was too weak to support the weight of the dynamometer, the patient was supported under the distal forearm. Two trials were performed for each muscle group on each side and the maximum value was used for analysis. Secondary endpoints included 6-min walk distance (6MWT), modified SMA function rating scale (SMA-FRS) score, Hammersmith Functional Rating Scale Expanded score (HFMSE), forced vital capacity (FVC), ulnar compound muscle action potential (CMAP) amplitude, ulnar single motor unit potential (SMUP) amplitude, and motor unit number estimation (MUNE) (18, 20, 22). Frequency and characteristics of clinically significant vital signs and laboratory abnormalities were assessed. Testing methodologies for strength measurement and functional assessments were consistent with prior published trials (18, 20). Ulnar CMAP was completed using standard techniques (http://smaoutcomes.org/hammersmith_manual/cmap.php), and multipoint incremental MUNE technique was used to obtain the SMUP and MUNE (22).

### Statistical Analysis

Linear mixed models were used to explore the change of outcome measures across time with random intercepts for each participant. Differences between each time point with baseline and 95% confidence intervals (CI) were reported. We performed a secondary analysis to compare longitudinal change over 10 years for outcome measures performed in 8 participants from the VALIANT study (18, 20). We used paired *t*-tests to compare measures of MVICT, SMA-FRS, 6MWT, and FVC. We presented *p*-values for all comparisons at the nominal level. Anonymized data analyzed for this study is available by request from qualified investigators.

## Results

### Baseline Characteristics and Nusinersen Tolerability

A total of 13 participants were enrolled and were assessed up to 14 months following treatment with nusinersen. Table 1 describes age, gender, baseline function, and SMN2 copies. The median age was 36.6 (range 18–58), and 7 (53.8%) were men. All patients have SMA type 3 except for one with SMA type 4. SMN2 copy dosage was as follows: 3 copies (4 participants), 4 SMN2 copies (8 participants), and 5 copies (1 participant). At baseline, 6MWT distance data available for 11 participants median was 297 m (range 107–495). Whereas, the baseline HFMSE median score available from all 13 participants was 49 (range 23–59), and the SMAFRS was 31 (range 26–36). Side effects (% of total injections) include headache (64%), low back pain (27%), nausea (14%), vomiting (8%), and dizziness (6%). The majority were described as mild and transient. Two participants had a more severe headache with nausea and vomiting that responded to conservative treatment. None needed a blood patch. None of the participants had clinically significant abnormalities of vital signs or laboratory parameters including CBC, coagulation profile or urine protein/creatinine ratio and no participants discontinued treatment. All participants reached at least the 10-month visit, and 10 were assessed at 14 months.

**Table 1:** Demographics and baseline characteristics.

**Variable**	**Level**	**Total (*n* = 13)**
Age	Median (IQR) (min, max)	36.6 (33, 43) (18, 59)
Gender	Women	6 (46.2%)
	Men	7 (53.8%)
Baseline 6-min walk distance (meters)	Median (IQR) (min, max)	297 (203, 387) (107, 495)
Baseline SMA-FRS (normal range 0–40)	Median (IQR) (min, max)	31 (29, 34) (26, 36)
Baseline HFMSE (normal range 0–66)	Median (IQR) (min, max)	49 (45, 52) (23, 59)
SMN2 copies	3	4 (30.8%)
	4	8 (61.5%)
	5	1 (7.7%)

### Prospective Outcome Assessments

Baseline values for outcome assessments and prospective comparisons at 2, 6, 10, and 14 months are show in Table 2. Pre-treatment baseline 6MWT and SMUP and MUNE assessments were missing for two participants. MVICT demonstrated no change at any timepoint. In contrast, both HFMSE and SMA-FRS both showed improvements at all timepoints. An increase in score by 3 in the HFMSE (maximum score 66) and an increase of 2 for the modified SMAFRS (maximum score 40) are considered clinically meaningful (23–25). When we compared the baseline pre-treatment score to the last assessment visit (10 or 14 months), the HFMSE score changed in the range of −3 to +8 points. A score increase by ≥3 points in HFMSE was seen in 5 (38%) and ≥0 points in 12 (92%). The modified SMAFRS score changed in the range of −1 to +5 points. A score increase by ≥2 points in modified SMAFRS score was seen in 6 of 13 (46%) and ≥0 points in 9 (69%). A decline of one point on the SMAFRS was noted in 4 participants. FVC demonstrated improvements at 6, 10, and 14 months; however, the changes were minimal. 6MWT was increased at 2, 6, 10, but not 14 months. Generally, a change of 30 m in the 6-min walk is considered clinically meaningful (25). The 6MWT distance changed in the range of −40 to +94 m. In 2 of 11 (18%) participants, an increase of ≥30 m for the 6MWT was seen, in 9 of 11 (81%) the 6MWT was stable or increased (≥0 m), and in one participant the 6MWT distance decreased by >30 m (40 m reduction). Increases in CMAP (6, 10, and 14 months) and SMUP (2, 10, and 14 months) were observed, but MUNE was unchanged.

**Table 2:** Longitudinal change of prospective outcomes following nusinersen treatment.

**Measure**	**Time/comparison**	***N***	**Estimate (baseline/change from baseline)^**#**^**	**95% CI**	***p*-value**
**Primary outcome**					
MVICT (Kg)	Baseline	13	6.75	4.3, 9.19	
	2 months—baseline	13	0.36	−0.2, 0.92	0.2051
	6 months—baseline	13	0.29	−0.27, 0.85	0.3057
	10 months—baseline	13	0.52	−0.04, 1.08	0.0672
	14 months—baseline	9	0.2	−0.43, 0.84	0.5222
**Functional scales**					
HFMSE	Baseline	13	45.85	40.41, 51.28	
	2 months—baseline	13	2.77	0.84, 4.69	0.0058
	6 months—baseline	13	2.46	0.54, 4.39	0.0134
	10 months—baseline	13	2.08	0.15, 4.00	0.0351
	14 months—baseline	10	2.6	0.5, 4.69	0.0165
SMA-FRS	Baseline	13	31.23	29.47, 32.99	
	2 months—baseline	13	1.69	0.42, 2.96	0.0102
	6 months—aseline	13	1.77	0.5, 3.04	0.0074
	10 months—baseline	13	1.23	−0.04, 2.50	0.0575
	14 months—baseline	11	1.57	0.23, 2.92	0.0224
**Physical function**					
Six minute walk test (meters)	Baseline	11	262.88	171.42, 354.35	
	2 months—baseline	13	19.49	2.91, 36.07	0.0223
	6 months—baseline	13	25.27	8.69, 41.85	0.0037
	10 months—baseline	13	19.19	2.61, 35.77	0.0243
	14 months—baseline	10	15.88	−2.16, 33.92	0.0829
Forced vital capacity (liters)	Baseline	13	4.07	3.38, 4.77	
	2 months—baseline	13	0.07	−0.04, 0.18	0.2206
	6 months—baseline	13	0.16	0.04, 0.27	0.0081
	10 months—baseline	13	0.12	0.01, 0.24	0.0332
	14 months—baseline	10	0.17	0.05, 0.3	0.0072
**Electrophysiological measures of motor unit connectivity**					
Compound muscle action potential amplitude (millivolts)	Baseline	13	8.42	7.16, 9.68	
	2 months—baseline	13	0.08	−0.22, 0.39	0.5766
	6 months—baseline	13	0.35	0.05, 0.66	0.0231
	10 months—baseline	13	0.41	0.10, 0.71	0.0095
	14 months—baseline	10	0.47	0.14, 0.80	0.0060
Average single motor unit potential amplitude (microvolts)	Baseline	11	80.83	71.14, 90.51	
	2 months—baseline	13	5.13	0.35, 9.91	0.0359
	6 months—baseline	13	3.76	−1.02, 8.55	0.1196
	10 months—baseline	13	6.69	1.91, 11.47	0.0072
	14 months—baseline	10	8.93	3.73, 14.13	0.0012
Motor unit number estimation	Baseline	11	106.17	88.14, 124.21	
	2 months—baseline	13	−2.17	−7.83, 3.48	0.4419
	6 months—baseline	13	−1.64	−7.29, 4.01	0.5623
	10 months—baseline	13	−2.48	−8.13, 3.17	0.3806
	14 months—baseline	10	−5.22	−11.37, 0.93	0.0940

# *Positive values indicate increase compared to baseline, and negative values indicate decrease from baseline at each time point (2, 6, 10, and 14 months)*.

### Long-Term Assessment of Decline of SMA Outcomes Over a 10-Year Period

No prior studies have investigated the long-term change in standard SMA outcome measures, and therefore, in 8 participants, outcomes previously assessed as part of our single-center VALIANT study were compared to the baseline/screening outcome assessments of the current study to assess change over a ten-year period (20). MVICT, SMA-FRS, 6MWT, and FVC showed significant declines (Table 3 and Supplementary Figure 1). However, there was no significant decline in CMAP. Two participants lost ambulation over the preceding 10 year period and were not enrolled in this study, but the remaining six who continued to be ambulatory were included in this open label, observational study.

**Table 3:** Ten-year change of outcome measures from eight participants in the prior VALIANT Study.

	**Ten years ago mean (SD)**	**Current mean (SD)**	**Difference (95% CI)^**#**^**	***p*-value**
MVICT (Kg)	7.34 (6.28)	5.67 (5.57)	−1.67 (−2.73, −0.62)	0.0073
SMA-FRS	33.38 (3.25)	28.75 (4.56)	−4.63 (−8.57, −0.68)	0.0276
6MWT (meters)	275.57 (157.11)	195 (177.56)	−80.57 (−131.1, −30.07)	0.0079
FVC (liters)	4.4 (0.94)	3.88 (0.85)	−0.52 (−0.96, −0.09)	0.0248
CMAP amplitude (millivolts)	7.9 (2.28)	6.54 (3.19)	−1.36 (−2.86, 0.13)	0.0679

# *Negative values indicate reduction in outcomes compared to measures obtained 10 years prior*.

## Discussion

Our study demonstrated that nusinersen had a favorable safety profile, was well-tolerated, and had a positive impact on the majority of outcomes that were assessed. These findings are similar to the largest studies to date in adults with SMA reported by Hagenacker et al. (11) and by Maggi et al. (17). The findings in our series add to the body of emerging literature supporting nusinersen treatment in adults with SMA (10–17). None of our patients experienced the rare serious side effects raised by recent reports (26). Our study is unique in that it offers insight into additional outcome measures [strength measurement and patient-reported outcome measure (modified SMAFRS)] as well as physiological mechanisms of effect of nusinersen on motor unit function.

### Lack of Significant Effect of Nusinersen on Primary Outcome (MVICT)

In our study, we chose MVICT as the primary outcome based on our prior experiences with various outcome measures in ambulatory adults with SMA (18, 20). MVICT, as compared to 6MWT, demonstrated superior test–retest reliability and tighter associations with other neuromuscular function outcomes and biomarkers in a cohort of ambulatory adults with SMA (18). We had previously shown that strength was stable on MVICT assessment over 12 months (20). In contrast to other functional outcome measures, MVICT did not demonstrate significant change in response to nusinersen. There are a few possible explanations for the discrepancies between the other functional outcomes and MVICT. One possible explanation might include the limited number of muscles that were assessed with MVICT, the fact that the most proximal muscles (shoulder abductors and hip flexors) were not assessed, and the small sample size. Interestingly, we have identified defects of neuromuscular junction transmission on repetitive nerve stimulation in patients with SMA despite chronic treatment with nusinersen (unpublished observation). As such, measures that assessed the ability to sustain force production over time (i.e., muscle power) as compared with peak isometric force may provide more sensitive assessments. Development of more optimal and sensitive methods for assessing muscle strength and function deserves more attention in future SMA clinical studies.

### Impact of Nusinersen on Secondary Outcome Measures

Our study showed significant increases in the HFMSE with a 2.6 point increase at 14 months, and 38% of the cohort showed at least a 3 point increase which has been defined as a clinically meaningful change (23). Furthermore, the majority of patients (92%) showed stability or slight increase which is in contrast to the expected decline in untreated patients of ≥0.5 points per year (19). Our findings are aligned with those noted in the study by Hagenacker et al., in which HFMSE score change for 35 ambulant subjects showed a mean difference from baseline of 4.3 ± 3.7 at 10 months and 4.6 ± 4.4 at 14 months (11). They reported an improvement of ≥3 points in HFMSE seen in 33 of 92 (35%) at 10 months and in 23 of 57 (50%) at 14 months (11). Similarly, the study by Maggi et al. reported a mean change among SMA type 3 walkers in the HFMSE was 2.38 ± 2.71 (median 2, range −3 to 8) in 40 subjects at 10 months and 2.37 ± 2.22 (median 2, range −2 to 6) in 27 subjects at 14 months (17). The percent increase in HFMSE of ≥3 points was 43 and 48% after 10 and 14 months of treatment (17).

In our study, the impact of nusinersen on the 6MWT was less robust as compared with the studies by Hagenacker et al. (11) and Maggi et al. (17). We found mean changes of 19.19 m at 10 months and 15.88 m at 14 months. The data by Hagenacker et al. on the 6MWT data showed 37 subjects treated with nusinersen had a mean difference at 10 months from baseline of 31.1 and 46 m at 14 months for 25 subjects (11). Maggi et al. reported a mean change in the 6MWT of 26.45 m in 35 subjects at 10 months and 23.11 m in 24 subjects at 14 months (17). An increase of at least 30 m is considered clinically meaningful for the 6MWT, and in the study by Maggi et al. this threshold was met by 46 and 42% of patients after 10 and 14 months of treatment, respectively (17, 24). This finding contrasts with the result in our cohort, with only 2 of 11 (18%) of our patients with baseline data met the threshold increase of 30 or more meters. This could be related to the small number of participants in the current study. This may also be related to a lower baseline score in our cohort of 297 compared to 322 in the Maggi trial though the difference of baseline values is not greater than the meaningful difference of at least 30 m (17). However, determining clinical meaningfulness based on an absolute value has its shortcomings (27). Contrasting the change to the baseline value could provide additional information. In our cohort, the two subjects who met the 30 m threshold represented an increase of 17 and 23% compared to their baseline. All the other patients in our cohort except for two had a positive change ranging from 2 to 20%. This contrasts with the natural history of the expected decline by 9.7 m per year (24).

We observed heterogeneity of nusinersen treatment response. We observed that the most significant improvements in the 6MWT were seen in two patients with high baseline values of 387 of 404 m, but the most significant improvements in HFMSE score (increase of 5 or more) were seen in patients with a range of baseline scores of 23, 32, 45, and 48. Maggi et al. found the most significant improvement in patients with worse baseline performance 6MWT (200 m or less), whereas, Hagenacker et al. found a higher efficacy of Nusinersen in patients with higher baseline HFMSE scores of 35 or more (11, 17). Additional work is needed to determine factors that may predict response.

In our study, similar to the HFMSE, the patient-reported outcome (modified SMAFRS) score increase by two or more points was noted in 38%. Overall, HFMSE and SMA-FRS appear to be sensitive measures of therapeutic response among ambulatory adult patients. SMA-FRS has added benefits of ease of assessment and thus could represent a pragmatic method to track response to treatment. Similar to other studies, we noted a trend of positive impact on the FVC, although minimal, is of unclear clinical impact given the normal lung function at baseline (10, 17).

### Physiological Impact of Nusinersen on Motor Unit Function

One of the main goals of the current study was to investigate the physiological impact of late SMN treatment on motor unit function. In younger patients, nusinersen has been shown to improve CMAP amplitude (16). However, the effects of SMN restoration in adults with SMA was unknown. Here we demonstrate the novel and previously unreported findings that nusinersen resulted in a progressive increase of CMAP in ambulatory adults with SMA. Similarly, we also investigated average motor unit size, or SMUP amplitude. CMAP is a non-specific electrophysiological readout that may reflect changes of the motor neuron, axon, neuromuscular junction or muscle fiber integrity. The parallel increases in CMAP and SMUP indicated motor unit enlargement suggesting improved collateral sprouting with nusinersen. Pre-clinical studies in mouse and pig models of SMA have shown that motor unit numbers are preserved with early but not delayed SMN restoration (28–30). Similar to these prior pre-clinical studies investigating late SMN restoration, MUNE was unchanged in the current study (28–30). Therefore, in our study nusinersen increased motor unit function but not number in adults with SMA. In a parallel study in non-ambulatory adults with SMA, we found a similar pattern of increases in CMAP and SMUP and stable MUNE (31). Interestingly, in a recent study that used MscanFit MUNE to estimate motor unit number in children with SMA treated with nusinersen, it was shown that nusinersen induced recovery of smaller motor units (32). Whether these differences are related to variability of biological response in children vs. adults or are explained by differences in technique requires further research.

### Loss of Muscle Strength, Motor Function, and Motor Unit Health in Ambulatory SMA Patients Over a 10-Year Period

Another goal of the current study was to investigate the decline of standard outcome measures and functional measures over a 10-year period in 8 participants studied longitudinally. These results demonstrated significant decline in strength, motor function and motor unit function and suggested that stability of function may also be a reasonable treatment goal with later SMN restoration.

### Conclusion

While the small sample size, open label design and limited longitudinal data may restrict generalizability of the findings, our data support the safety and efficacy of nusinersen in the treatment of ambulatory adults with SMA and suggest its positive effect occurs *via* improved motor unit size.

## Data Availability Statement

The original contributions presented in the study are included in the article/Supplementary Material, further inquiries can be directed to the corresponding author.

## Ethics Statement

The studies involving human participants were reviewed and approved by The Ohio State University. The patients/participants provided their written informed consent to participate in this study.

## Author Contributions

BE: drafting/revision of the manuscript for content, including medical writing for content, major role in the acquisition of data, study concept or design, and analysis or interpretation of data. SS, SZ, DK, SK, and WA: drafting/revision of the manuscript for content, including medical writing for content, study concept or design, and analysis or interpretation of data. ML, MT, AB, SH, and GS: drafting/revision of the manuscript for content, including medical writing for content, and major role in the acquisition of data. KK: drafting/revision of the manuscript for content, including medical writing for content, major role in the acquisition of data, and analysis or interpretation of data. TW and KR: drafting/revision of the manuscript for content, including medical writing for content, and analysis or interpretation of data. All authors contributed to the article and approved the submitted version.

## Conflict of Interest

BE received compensation for consulting from Biogen, Genentech, Argenx, and Stealth Bio-therapeutics. TW received compensation for consulting from Medtronic, Inc. and PainTeq. SK received compensation for consulting from Genentech, AveXis and Biogen. WA received compensation for consulting for La Hoffmann Roche and Genentech. The remaining authors declare that the research was conducted in the absence of any commercial or financial relationships that could be construed as a potential conflict of interest.
