# Supplementary material for: Safety, Tolerability, and Effect of Nusinersen Treatment in Ambulatory Adults With 5q-SMA
Source: Front Neurol. 2021 May 20;12:650535. doi: 10.3389/fneur.2021.650535 (PMC8174580; doi:10.3389/fneur.2021.650535)

Supplemental Figure 1: Longitudinal Plots of Participants studied 10 years prior to baseline.

A. Maximum Voluntary Isometric Contraction Testing (MVICT) Sum Score

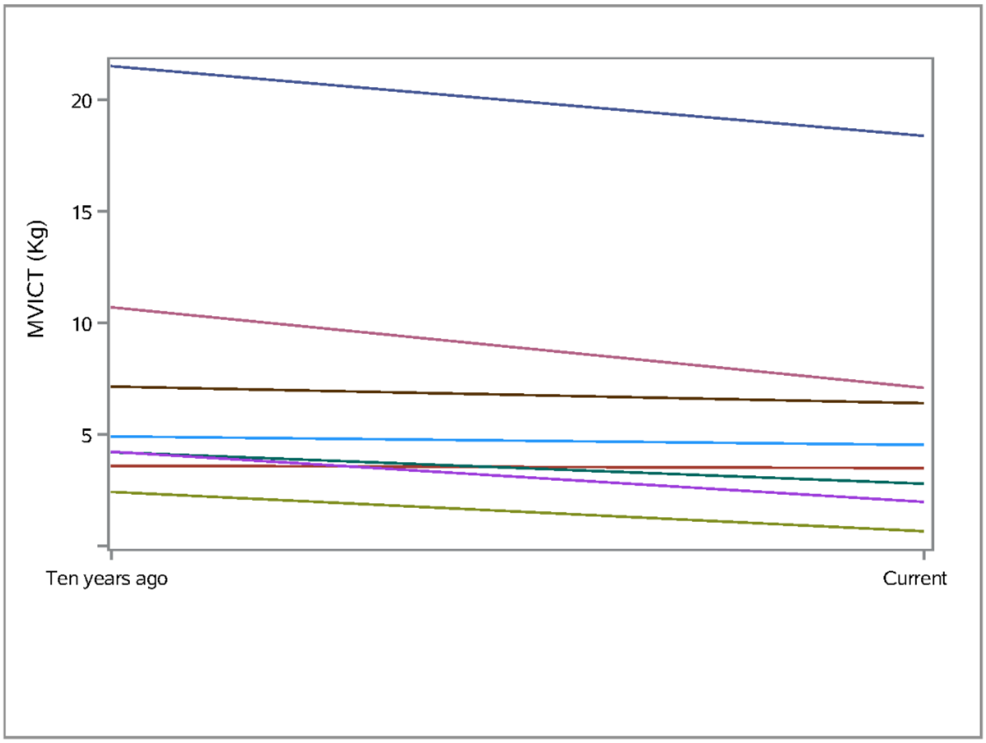

B. Spinal muscular atrophy functional rating scale (SMA-FRS)

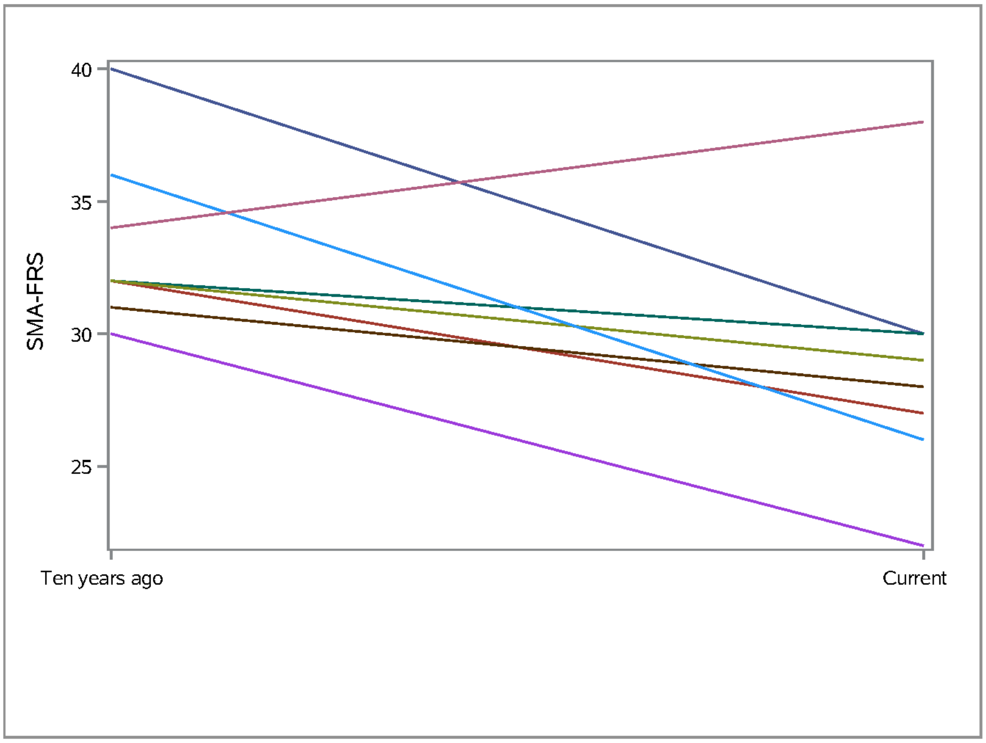

C. Six minute walk test (6MWT) Distance (meters)

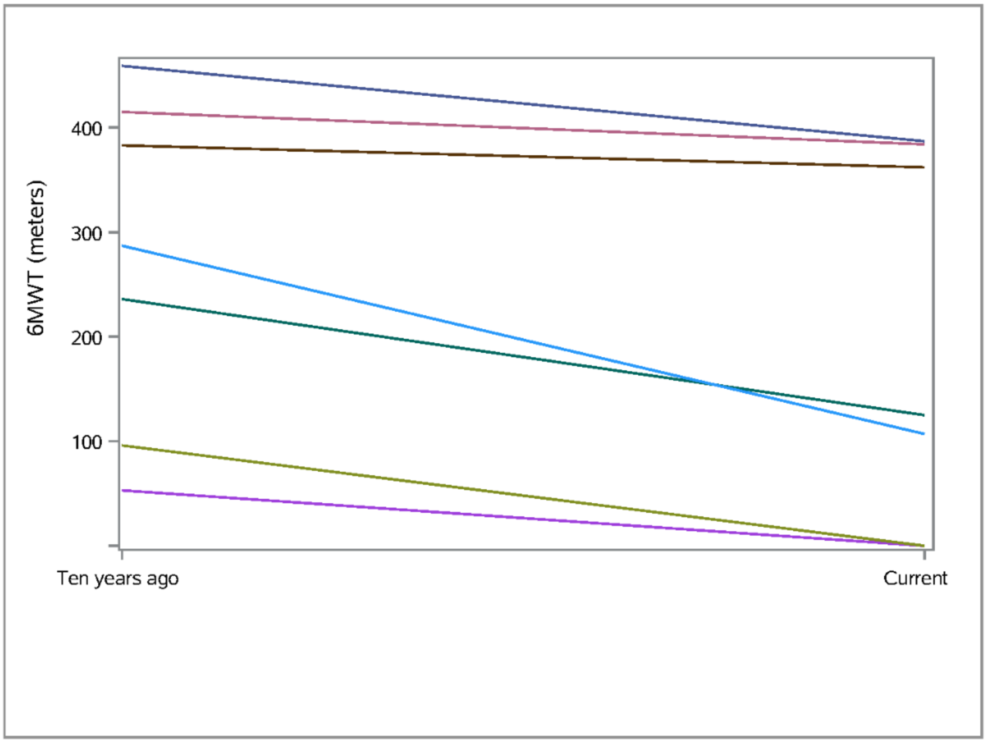

D. Forced vital capacity (liters)

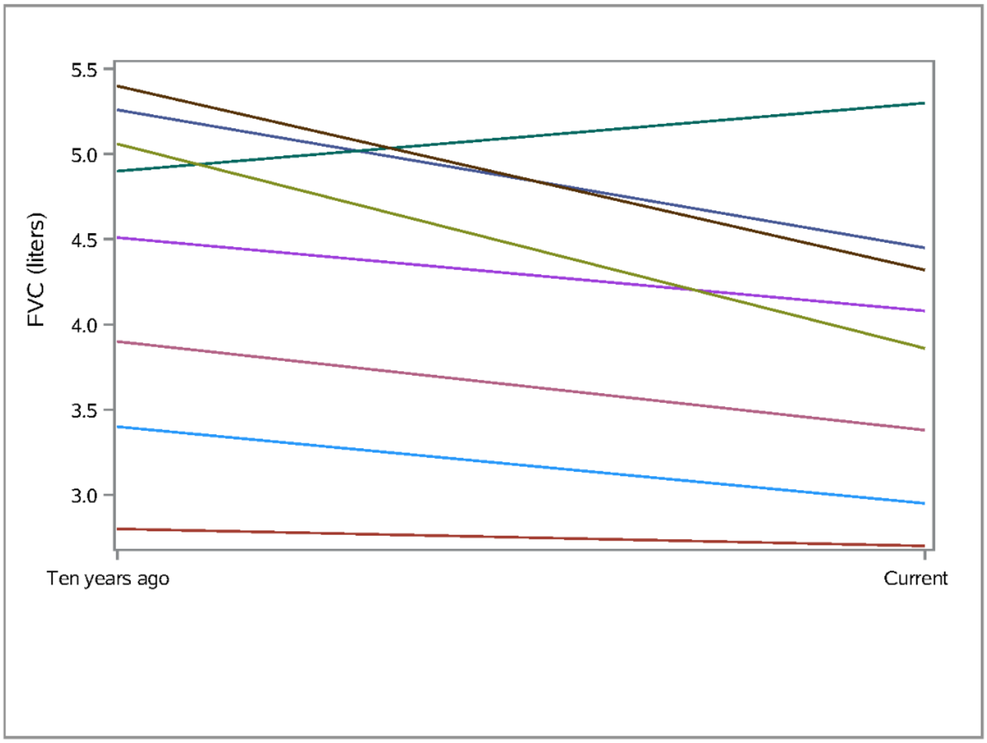

E. Compound muscle action potential (CMAP) Amplitude

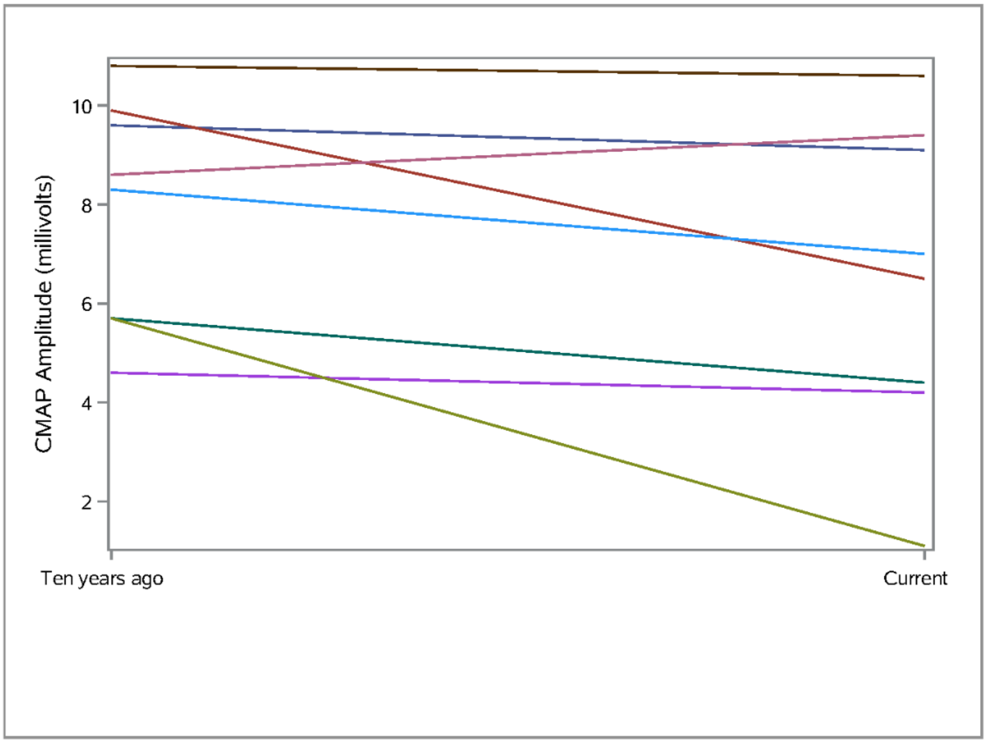

Supplement: Supplementary file 1 [file Image_1.PDF]
